# Supplementation with high-GABA-producing *Lactobacillus plantarum* L5 ameliorates essential tremor triggered by decreased gut bacteria-derived GABA

**DOI:** 10.1186/s40035-023-00391-9

**Published:** 2023-12-13

**Authors:** Hao-Jie Zhong, Si-Qi Wang, Ruo-Xin Zhang, Yu-Pei Zhuang, Longyan Li, Shuo-Zhao Yi, Ying Li, Lei Wu, Yu Ding, Jumei Zhang, Xinqiang Xie, Xing-Xiang He, Qingping Wu

**Affiliations:** 1 Guangdong Provincial Key Laboratory of Microbial Safety and Health, State Key Laboratory of Applied Microbiology Southern China, Institute of Microbiology, Guangdong Academy of Sciences, Guangzhou, 510000 China; 2 Department of Gastroenterology, the First Affiliated Hospital of Guangdong Pharmaceutical University, Guangzhou, 510000 China

**Keywords:** Cerebellum, Essential tremor, Gamma-aminobutyric acid, Gut microbiota, *Lactobacillus plantarum*

## Abstract

**Background:**

The γ-aminobutyric acid (GABA) hypothesis posits a role of GABA deficiency in the central nervous system in the pathogenesis and progression of essential tremor (ET). However, the specific causative factor for GABA deficiency is not clear. The gut microbiota in mammals has recently been considered as a significant source of GABA. Furthermore, the GABA-based signals originating from the intestine can be transmitted to the brain through the “enteric nervous system–vagus nerve–brain” axis. However, the plausible contribution of gut microbiota to ET seems inspiring but remains obscure.

**Methods:**

Fecal samples from patients with ET and healthy controls were examined by metagenomic sequencing to compare the composition of gut microbiota and the expression of genes involved in GABA biosynthesis. The impact of gut microbiota on ET was explored through transplantation of fecal microbiota from patients with ET into the murine ET model. Lactic acid bacteria producing high amounts of GABA were identified through whole-genome sequencing and ultra-performance liquid chromatography-tandem mass spectrometry. Subsequently, mice were treated with the high-GABA-producing strain *Lactobacillus plantarum* L5. Tremor severity, behavioral tests, pro-inflammatory cytokines, GABA concentration, and gut microbiota composition were examined in these mice.

**Results:**

The gut microbiota of patients with ET demonstrated an impaired GABA-producing capacity and a reduced fecal GABA concentration. Transplantation of the gut microbiota from patients with ET induced an extension of tremor duration and impaired mobility in the murine model of ET. L5 exhibited an augmented GABA-producing capacity, with the De Man-Rogosa-Sharpe culture broth containing 262 mg/l of GABA. In addition, administration of L5 significantly decreased the tremor severity and enhanced the movement capability and grasping ability of ET mice. In vivo mechanistic experiments indicated that L5 reshaped the gut microbial composition, supplemented the mucosa-associated microbiota with GABA-producing capacity, increased the GABA concentrations in the cerebellum, and diminished inflammation in the central nervous system.

**Conclusions:**

These findings highlight that deficiency of GABA-producing gut microbes plays an essential role in the pathogenesis of ET and that L5 is a promising candidate for treating ET.

**Supplementary Information:**

The online version contains supplementary material available at 10.1186/s40035-023-00391-9.

## Background

Essential tremor (ET) is the most common form of pathological tremor and one of the most prevalent movement disorders [1]. It is characterized by a postural and kinetic tremor primarily in the upper extremities [1, 2]. The global prevalence of ET is 3.2‰ overall, which increases to 28.7‰ in those aged over 80 [3]. The pathogenesis of ET remains incompletely understood, and only about half of the ET patients treated with first-line therapies exhibit a significant reduction in tremor amplitude [4, 5].

Mounting evidence suggests a link between ET and γ-aminobutyric acid (GABA), a major inhibitory neurotransmitter in the brain [6]. Decreased GABA in the cerebrospinal fluid has been found in patients with ET [7]. Additionally, ^11^C-flumazenil positron emission tomography studies indicated GABAergic dysfunction in the cerebellum of patients with ET [8, 9]. Moreover, the number of GABA receptors in the dentate nuclei of patients with ET is reduced and correlates with tremor severity [10]. Thus, GABA may be involved in the pathogenesis and progression of ET.

A recent study suggested that the gut microbial composition differs between patients with ET and healthy controls, indicating a link between gut microbiota and ET [11]. Additionally, a previous study by our group revealed that receiving a gut microbiota transplant from a healthy donor markedly attenuated the tremor in a patient with ET [12]. The gut microbiota is identified as an essential source of GABA in humans [13], suggesting that targeting the gut microbiota-dependent GABA productivity might be a novel therapy for ET. However, the association of the GABA-producing capacity of the gut microbiota with ET remains to be elucidated. Therefore, this study aimed to evaluate the characteristics and potential role of GABA-producing gut microbiota in the progression of ET.

## Methods

### Participants and sample collection

Patients with ET and healthy individuals (controls) from the First Affiliated Hospital of Guangdong Pharmaceutical University (Guangzhou, China) were eligible for inclusion in this study. Participants were excluded if they had any central nervous system (CNS) disease other than ET; any gastrointestinal illness such as cancer, peptic ulcer, or inflammatory bowel disease; severe pulmonary, cardiovascular, liver, or renal disorders. Participants who had been treated with antibiotics, probiotics, prebiotics, or fecal microbiota transplantation in the past three months before the study were also excluded. ET was diagnosed based on the criteria described by the Movement Disorder Society [14]. Fecal samples were collected from the ET patients and age- and sex-matched healthy controls and stored at − 80 ℃.

This study was conducted according to the Declaration of Helsinki and approved by the Research Ethics Board of the First Affiliated Hospital of Guangdong Pharmaceutical University (#2019–69-01). Informed consent was obtained from all subjects included in the study.

### Animals

Male C57BL/6 mice of 8–10 weeks old were purchased from the Guangdong Medical Laboratory Animal Center. The mice were housed under specific-pathogen-free (SPF) conditions with food and water ad libitum. The mice were acclimated for at least two weeks before conducting the experiments. All animal experiments were approved by the Animal Ethics Committee of the First Affiliated Hospital of Guangdong Pharmaceutical University (#201906001).

### Bacterial strains and growth conditions

In total, 572 strains of lactic acid bacteria (LAB) were isolated from the food and fecal samples collected previously and stored in the Laboratory of Food Safety and Health Development, Institute of Microbiology, Guangdong Academy of Sciences. All LAB were cultured in the De Man-Rogosa-Sharpe (MRS) broth (Guangdong HuanKai Microbial Sci. & Tech. Co. Ltd., Guangzhou, China) for 48 h at 37 °C in an anaerobic chamber (Don Whitley Scientific, Yorkshire, UK). The OD_600_ of each culture was measured to determine the growth state suitable for use.

### Mouse model of ET

Mice were intraperitoneally injected with harmaline (8 mg/kg) to induce tremors. Successful construction of the murine ET model was verified by the tremor intensity score.

### Microbiota transplantation

Mice were administered with drinking water supplemented with 1 g/l ampicillin (XY Biotechnology, Shanghai, China), 0.5 g/l vancomycin (Shanghai Macklin Biotechnology Co. Ltd., Shanghai, China), 0.5 g/l neomycin (XY Biotechnology), 100 mg/l gentamycin (Solarbio, Beijing, China), and 10 mg/l erythromycin (Sigma Aldrich, St. Louis, MO) for two weeks before transplantation [15]. Fresh fecal samples, 100 g each from healthy controls (*n* = 5) or patients with ET (*n* = 5), were mixed with normal saline, homogenized, and microfiltered using a GenFMTer automatic machine (FMT Medical, Nanjing, China), as described previously [16]. The mixture was centrifuged at 1100 × *g* for 3 min, and the microbial pellet was then washed three times with saline. After that, the pellet was suspended in 100 ml of saline and stored with 50% glycerol at − 80 °C until further use. Prior to use, the microbial cells were thawed and rinsed with saline to remove the glycerol.

Mice were randomly divided into two groups (*n* = 15/group): (1) HC group, administered with 100 μl of the fecal suspension from healthy controls; and (2) ET group, administered with 100 μl of the fecal suspension from ET patients. The fecal suspension was administered using oral gavage, three times per week for three weeks. Thereafter, fecal samples from the test animals were collected, and behavioral tests (open-field and rotarod tests) were performed. The following day, the mice were intraperitoneally injected with harmaline to induce tremors, after which the behavioral tests were conducted. The entire experiment was performed twice independently.

### Oral treatment with GABA

Mice (*n* = 6/group) received drinking water or drinking water with 2 mg/ml GABA for seven days. Subsequently, tremors were induced through the intraperitoneal injection of harmaline, followed by an evaluation of tremor severity and duration.

### Probiotic treatment

Mice were randomly divided into two groups (*n* = 7 or 8/group): (1) NS group, administered with 200 μl of normal saline daily for four weeks; and (2) L5 group, administered with *L. plantarum* L5 (10^9^ CFUs/ml) daily for four weeks. After that, the mice were intraperitoneally injected with harmaline to induce tremors. After 15 min, behavioral tests such as the open-field, pole descent, grip strength, and rotarod tests were performed. Fecal samples were collected every 10 days from day 0 to day 40. Cerebellum, cerebrum, and plasma samples were collected on day 40. Additionally, 3-cm-long segments of colon and ileum were collected, washed with normal saline, and then swabbed for mucosa-associated microbes. These experiments were performed independently at least twice.

### Dead probiotic treatment

Mice were randomly assigned to two groups (*n* = 8/group): (1) NS group receiving daily administration of 200 μl of normal saline for four weeks; and (2) Dead L5 group, which received daily administration of 200 μl of a suspension containing dead L5 (10^9^ CFUs/ml) for four weeks. The dead L5 was prepared by heating the suspension at 100 ℃ for 20 min. Subsequently, the mice were intraperitoneally injected with 8 mg/kg of harmaline. After 15 min, behavioral tests such as open-field, pole descent, grip strength, and rotarod tests were performed.

### Behavioral tests

Tremor intensity was scored from 0 to 4 (0 = no tremor; 1 = mild tremor; 2 = moderate intermittent tremor; 3 = moderate persistent tremor; and 4 = pronounced severe tremor) [17] by two independent investigators who were blinded to the group allocation, and the two scores were averaged. The onset time was calculated as the time between harmaline injection and onset of visible tremors. Additionally, open-field [18], pole descent [19], grip strength [20], and rotarod [18] tests were performed, as previously described.

### Enzyme-linked immunosorbent assay (ELISA)

The levels of interleukin (IL)-1β (432604; BioLegend, San Diego, CA), IL-6 (431304; BioLegend), and tumor necrosis factor (TNF)-α (430904; BioLegend) in the mouse cerebellum and cerebrum were determined using sandwich ELISA kits according to the manufacturer’s instructions.

### Histological analyses

Mouse small intestines were fixed for 24 h in 4% paraformaldehyde (Beyotime Biotechnology, Shanghai, China), dehydrated, and embedded in paraffin. Hematoxylin and eosin staining was performed using 2-μm sections according to routine protocols. The slides were observed and photographed using a BX43 inverted microscope (Olympus, Shinjuku City, Tokyo, Japan).

### 16S rRNA gene sequencing and analysis

To investigate the effects of L5 on the gut microbiota of ET mice, 16S rRNA gene was sequenced using samples from ET mice treated with L5 or normal saline. DNA from the microbes of the fecal samples and intestinal mucosa was extracted using an E.Z.N.A.® Soil DNA Kit (Omega Bio-Tek Inc., Norcross, GA). The V3–V4 region of the 16S rRNA gene was amplified by PCR using the primers 338F (5′-ACTCCTACGGGAGGCAGCAG-3′) and 806R (5′-GGACTACHVGGGTWTCTAAT-3′) and the following program: 30 s at 95 °C, 30 s at 55 °C, and 45 s at 72 °C, for 27 cycles. Amplicons were sequenced on a PE300 Illumina MiSeq sequencing platform (Shanghai Majorbio Bio-Pharm Technology Co. Ltd., Shanghai, China).

Raw data were merged, filtered for quality, and denoised using the DADA2 pipeline available in QIIME2 to identify amplicon sequence variants (ASVs) [21, 22]. ASVs were assigned to taxa using the naïve Bayes consensus taxonomy classifiers implemented in QIIME2 and the SILVA 16S rRNA database v138. The sequencing data were analyzed using the Majorbio Cloud Platform (www.majorbio.com). Functional profiles of the microbial communities were predicted using PICRUSt2. The bacterial 16S rRNA gene sequencing data can be accessed from the SRA database (accession number PRJNA 904241).

### Metagenomic sequencing analysis

Metagenomic sequencing was performed to compare the composition of gut microbiota and the expression of their genes encoding key enzymes involved in GABA biosynthesis and degradation between patients with ET and healthy individuals. DNA was extracted from the microbes of the fecal samples and intestinal mucosa using an E.Z.N.A.® Soil DNA Kit (Omega Bio-Tek Inc., Norcross, GA). DNA concentration and purity were measured using a NanoDrop 2000 (Thermo Fisher Scientific, Waltham, MA), and integrity was assessed using 1% agarose gel electrophoresis. The DNA samples were then fragmented to a mean size of ~ 400 bp using an M220 Focused-ultrasonicator™ (Covaris, Brighton, UK). A sequencing library was prepared using the NEXTFLEX® Rapid DNA-Seq Kit (PerkinElmer Applied Genomics, Waltham, MA). Paired-end sequencing was performed using a NovaSeq System with NovaSeq Reagent Kits (Illumina Inc., San Diego, CA) according to the manufacturer’s instructions. The sequencing data were analyzed using the Majorbio Cloud Platform (www.majorbio.com). The metagenomic data can be accessed from the SRA database (accession number PRJNA 904560).

### Whole-genome sequencing analysis

Genomic DNA for whole-genome sequencing to detect glutamate decarboxylase (GAD)-encoding genes was extracted from 572 LAB strains using a Bacterial DNA Extraction Kit (Mabio), according to the manufacturer’s instructions. The sequencing libraries were prepared using an AMT Rapid DNA-Seq Kit (CISTRO) and sequenced on a NextSeq 550 platform (Illumina Inc.) using a High Output v2.5 Kit (Illumina Inc.). Sequencing reads were trimmed for the Illumina adapters using Trimmomatic v0.39, and then whole genomes were assembled using SPAdes v3.13.1. Subsequently, all genome assemblies were evaluated using Quast v5.0.2 and annotated using Prokka v1.13. Sequence alignment was performed using the Geneious software (Geneious).

### Ultra-performance liquid chromatography-tandem mass spectrometry

To identify the highest-GABA-producing strain of the 12 LAB strains with *GAD*, the strains were cultured in the MRS broth (Guangdong HuanKai Microbial Sci. & Tech. Co. Ltd.) for 48 h at 37 °C in an anaerobic chamber (Don Whitley Scientific). Each bacterial suspension was centrifuged at 12,000 rpm for 10 min at 4 °C, and the pellet was discarded. Supernatants were filtered through a 0.22-μm filter.

Chromatographic separation conditions: 4.6 mm × 250 mm × 5 μm Zorbax Sil column (Agilent Technologies Inc., Santa Clara, CA); mobile phases: A, acetonitrile and B, 0.1% aqueous formic acid solution; elution gradient: 0–0.5 min of 5% B, 0.5–4 min of 5%–30% B, 4–5.5 min of 30%–50% B, 5.5–7 min of 50% B, 7–9 min of 50%–95% B, and 9–10 min of 95% B; column temperature: 40 °C; injection volume: 2 μl; and flow rate: 0.3 ml/min.

Mass spectrometry conditions: triple quadrupole mass spectrometer (Agilent); drying gas: N_2_; drying gas flow rate: 11 l/min; drying gas temperature: 350℃; mass separation conditions: electrospray ionization in positive mode; capillary voltage: 4 kV; atomization pressure: 65 psi; and ionization voltage: 75 V. Selective reaction mode for GABA: collision energy at 8 V; precursor: 104.1 m/z; product: 87.1 m/z. Quantification was performed using a standard calibration curve plotted based on varying concentrations of GABA.

### Targeted metabolomics using liquid chromatography-tandem mass spectrometry

Targeted metabolomics was performed to determine the levels of the following neurotransmitters in ET mice treated with L5 or normal saline: 5-HIAA (5-hydroxyindole-3-acetic acid), 5-HT (serotonin hydrochloride), 5-HTP (5-hydroxytryptophan), Ach (acetylcholine chloride), DA (hydroxytyramine hydrochloride), DOPA (levodopa), E (adrenaline hydrochloride), GABA, Gln (l-glutamine), Glu (l-glutamic acid), His (L-histidine), HisA (histamine), Kyn (DL-kynurenine), KynA (kynurenic acid), MT (melatonine), NE (noradrenaline hydrochloride), PA (picolinic acid), Trp (L-tryptophan), TrpA (tryptamine), Tyr (L-tyrosine), TyrA (tyramine), VMA (vanillymandelic acid), and XA (xanthurenic acid). For this, ~ 50 mg of small intestine, cerebellum, or fecal sample was mixed with 600 μl of 10% methanol-formic acid solution and homogenized. The homogenates were centrifuged at 12,000 rpm for 10 min at 4 °C, and the pellet was discarded. Then 100 μl of the supernatant or plasma sample was mixed with 100 μl of 10 ppb Trp-d3 as an internal standard and passed through 0.22-μm filters.

Chromatographic separation conditions: 4.6 × 150 mm Zorbax Eclipse XDB-C18 column (Agilent); mobile phases: A, 10% methanol + 90% water containing 0.1% formic acid and B, 50% methanol + 50% water containing 0.1% formic acid; elution gradient: 0–6.5 min of 10%–30% B, 6.5–7 min of 30%–100% B, 7–18 min of 100% B, 18–18.5 min of 100%–10% B, 18.5–21 min of 10% B; column temperature: 40 ℃; injection volume: 5 μl; flow rate: 0.3 ml/min for 0–8 min and 0.4 ml/min for 8.5–21 min.

Mass separation conditions were: electrospray ionization in a positive mode; ion source temperature: 500 °C; ion source voltage: 5500 V; collision gas: 6 psi; curtain gas: 30 psi; nebulizer gas: 50 psi; and auxiliary gas: 50 psi. Multiple reaction monitoring mode detected the targeted metabolites (Additional file 1: Table S1). Quantification was performed using calibration curves plotted based on the relevant standards.

### Statistical analysis

Data are expressed as the mean ± standard deviation or median with interquartile range. The statistical significance of the differences between the groups was assessed using an independent Student’s *t*-test or Wilcoxon rank-sum test. Correlation analyses were performed using Spearman’s rank correlation. *P* < 0.05 was considered as statistically significant. The experimental data were analyzed using GraphPad Prism v8.0.2 (GraphPad Software Inc.) and the Wekemo Bioincloud platform (www.bioincloud.tech).

## Results

### Gut microbiota from patients with ET demonstrated a lower GABA-producing capacity

**Fig. 1:**
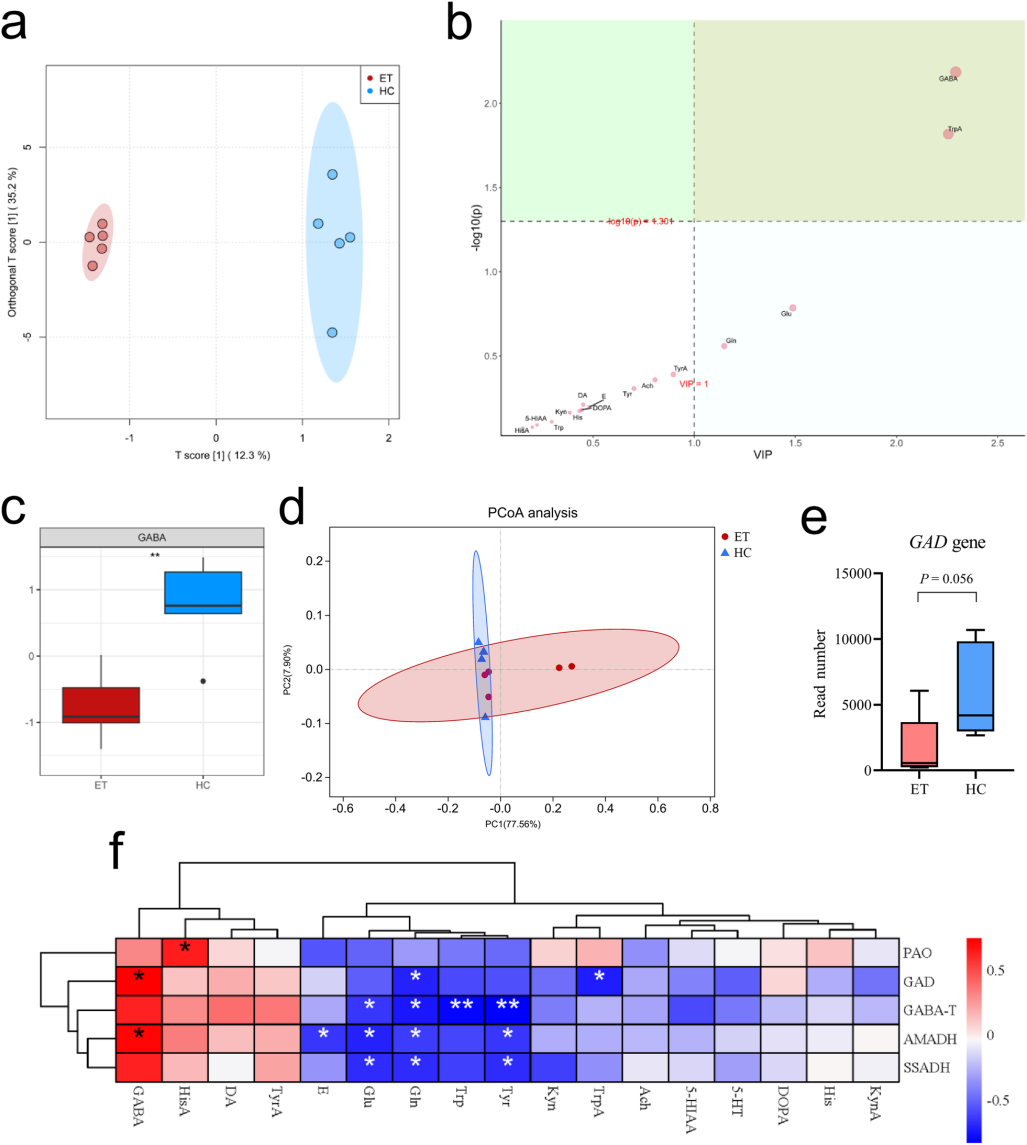
Gut microbiota from patients with ET exhibited a lower GABA-producing capacity. **a** Orthogonal partial least-squares discriminant analysis (OPLS-DA) score plot of fecal neurotransmitter levels in patients with ET and healthy individuals (controls). **b** Important neurotransmitters (GABA and TrpA) identified by OPLS-DA based on variable importance in projection (VIP) scores. **c** Relative levels of fecal GABA. **d** Principal coordinate analysis (PCoA) of microbiota composition at the generic level.** e** Expression of *GAD* (glutamate decarboxylase) in gut microbiota. **f** Heatmap indicating the correlations between the expression levels of genes encoding GABA-related enzymes and fecal neurotransmitter levels. 5-HIAA, 5-hydroxyindole-3-acetic acid; 5-HT, serotonin hydrochloride; Ach, acetylcholine chloride; AMADH, γ-aminobutyraldehyde dehydrogenase; DA, hydroxytyramine hydrochloride; DOPA, levodopa; E, adrenaline hydrochloride; ET, essential tremor; GABA, γ-aminobutyric acid; GABA-T, γ-aminobutyric acid transaminase; GAD, glutamate decarboxylase; Gln, L-glutamine; Glu, L-glutamic acid; HC, healthy controls; His, L-histidine; HisA, histamine; Kyn, DL-kynurenine; KynA, kynurenic acid; PAO, polyamine oxidase; SSADH, succinic semialdehyde dehydrogenase; Trp, L-tryptophan; TrpA, tryptamine; Tyr, L-tyrosine; TyrA, tyramine. Box plots indicate the median and interquartile range. ^*^P < 0.05; ^**^P < 0.01

The levels of various neurotransmitters in the fecal samples were compared between ET patients (*n* = 5) and age- and sex-matched healthy controls (*n* = 5) (Additional file 1: Tables S2 and S3). An orthogonal partial least-squares discriminant analysis (OPLS-DA) of the selected metabolomics data revealed that the fecal neurotransmitter levels varied markedly between the two groups (Fig. 1a). After calculating the variable importance in projection (VIP) value, GABA and tryptamine were identified to be the critical neurotransmitters with differential abundance between the two groups with VIP > 1 and *P* < 0.05 (Fig. 1b). Further quantitative analysis revealed a conspicuous inhibition in the fecal GABA levels, which emerged as the most prominently altered neurotransmitter in patients with ET (Fig. 1c), indicating that reduced levels of GABA might be involved in the pathogenesis of ET.

As various gut microbes are capable of GABA synthesis or catabolism [23], metagenomic sequencing was performed to compare the composition of gut microbiota and their expression of key genes encoding enzymes involved in the biosynthesis and degradation of GABA by the gut microbiota between patients with ET and healthy individuals. The GABA-biosynthesis-related enzymes were GAD, AMADH (γ-aminobutyraldehyde dehydrogenase), and PAO (polyamine oxidase), while the degradation-associated enzymes were GABA-T (GABA transaminase) and SSADH (succinic semialdehyde dehydrogenase). Although the principal coordinate analysis (PCoA) did not find any significant differences in the composition of gut microbiota (Fig. 1d), decreased expression of *GAD* was detected in the gut microbiota of the patients with ET (*P* = 0.056) (Fig. 1e). Moreover, *GAD* expression was positively correlated with fecal GABA levels (Fig. 1f), demonstrating that fecal GABA may be predominantly produced by GABA-producing gut microbes expressing *GAD*. In summary, these results suggested that the gut microbiota in patients with ET possessed a lower GABA-producing capacity and may participate in the development of ET.

### Gut microbiota derived from patients with ET extended the duration of tremors and compromised the mobility of ET mice

**Fig. 2:**
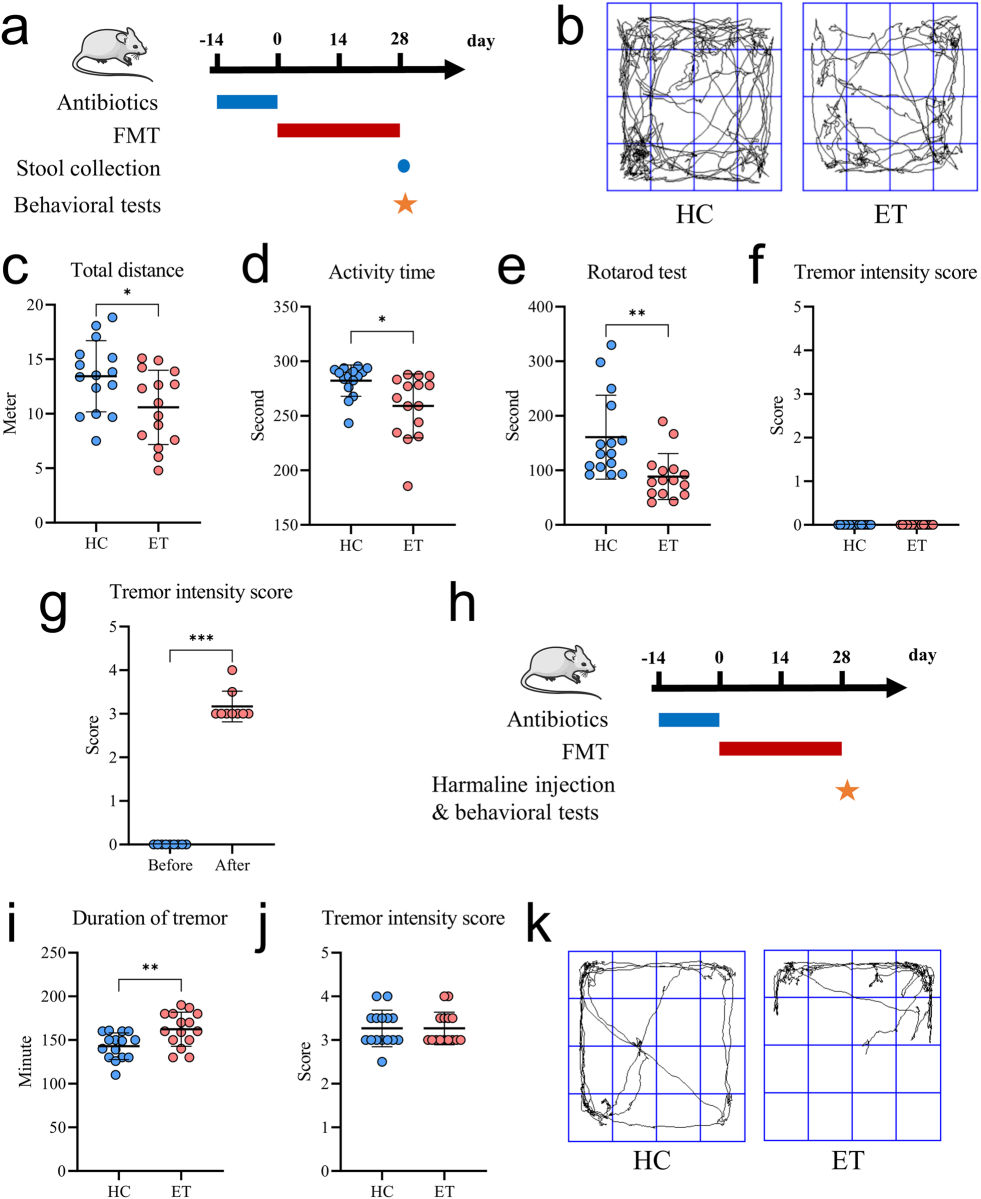
Gut microbiota derived from patients with ET extended the duration of tremors and compromised mobility in mice models of ET. **a** Study design involving the transplantation of gut microbiota from ET patients and healthy controls into mice (three times per week for three weeks). **b** Representative activity trajectories of mice receiving fecal microbiota from ET patients and healthy controls. **c**-**f** Total distance covered (**c**), active duration in 5 min (**d**), duration of rotarod test (**e**), and tremor intensity score (**f**) of mice receiving gut microbiota from ET patients or healthy controls. **g** Tremor intensity score in mice before and after receiving intraperitoneal injection of harmaline. **h** Study design involving mice treated with gut microbiota from ET patients and healthy controls, followed by harmaline treatment to induce ET. **i**-**k** Duration of tremor (**i**), tremor intensity score (**j**), and representative activity trajectories (**k**) of mice receiving fecal microbiota from ET patients and healthy controls after harmaline injection. HC, healthy controls; ET, essential tremor; FMT, fecal microbiota transplantation. Data are presented as the mean ± standard deviation. ^*^P < 0.05; ^**^P < 0.01

Next, to determine whether low-GABA-producing microbes contributed to the pathogenesis and development of ET, the gut microbiota from ET patients and healthy controls were transplanted into the SPF mice (Fig. 2a). Three weeks after transplantation, a notable disparity in the composition of gut microbiota was observed between the two groups (Additional file 1: Fig. S1a). Additionally, the mice receiving gut microbiota of ET patients (ET group) exhibited a considerably diminished relative abundance of microbiota that expressed *GAD* compared to those receiving HC gut microbiota (Additional file 1: Fig. S1b). Moreover, the members of the ET group exhibited several behavioral abnormalities, including a decreased total distance moved, reduced activity time, and impaired motor function (Fig. 2b–e). However, visible tremor was not observed in either the ET or the HC group (Fig. 2f).

Following the intraperitoneal injection of 8 mg/kg harmaline into nine healthy mice, tremors were conspicuously observed, indicating the successful establishment of the murine ET model (Fig. 2g). Subsequently, the mice receiving ET or HC gut microbiota transplantation were intraperitoneally injected with harmaline, after which the behavioral tests were conducted (Fig. 2h). Although no notable difference was observed in the tremor intensity between the two groups, the ET group exhibited a prolonged duration of tremor and impaired mobility compared to the HC group (Fig. 2i–k). These findings revealed that the low-GABA-producing gut microbes were responsible for an extended duration of tremor and compromised mobility in the mice with ET, suggesting that the gut microbiota may serve as a therapeutic target for ET.

### High-GABA-producing *L. plantarum* L5 ameliorated the harmaline-induced ET symptoms in mice

Despite the significant increase in plasma GABA concentrations from oral treatment [24], only minimal quantities can traverse the blood–brain barrier [25]. In this study, we also found that the oral administration of GABA failed to alleviate the severity or duration of tremors in murine ET (Additional file 1: Fig. S2).

Considering the substantial transmission of signals involving GABA between the gut and the brain via the “enteric nervous system–vagus nerve–brain” axis [25], direct delivery of GABA to the intestine may be more efficient in treating ET. Since several species of LAB can produce GABA and colonize the intestine [26, 27], LAB strains isolated from food and fecal samples were screened for GABA-producing capacity. An experiment was then performed to investigate whether administering a high-GABA-producing strain could alleviate the tremor in ET mice.

Of the 572 LAB strains screened, 12 contained the GAD-encoding genes *GadA* and *GadB*, as detected by whole-genome sequencing. Further ultra-performance liquid chromatography-tandem mass spectrometry analysis revealed that the *L. plantarum* strain L5 exhibited the highest GABA-producing capacity compared to other LAB strains. Specifically, the MRS broth of L5 contained 262 mg/l of GABA (Additional file 1: Table S4).

**Fig. 3:**
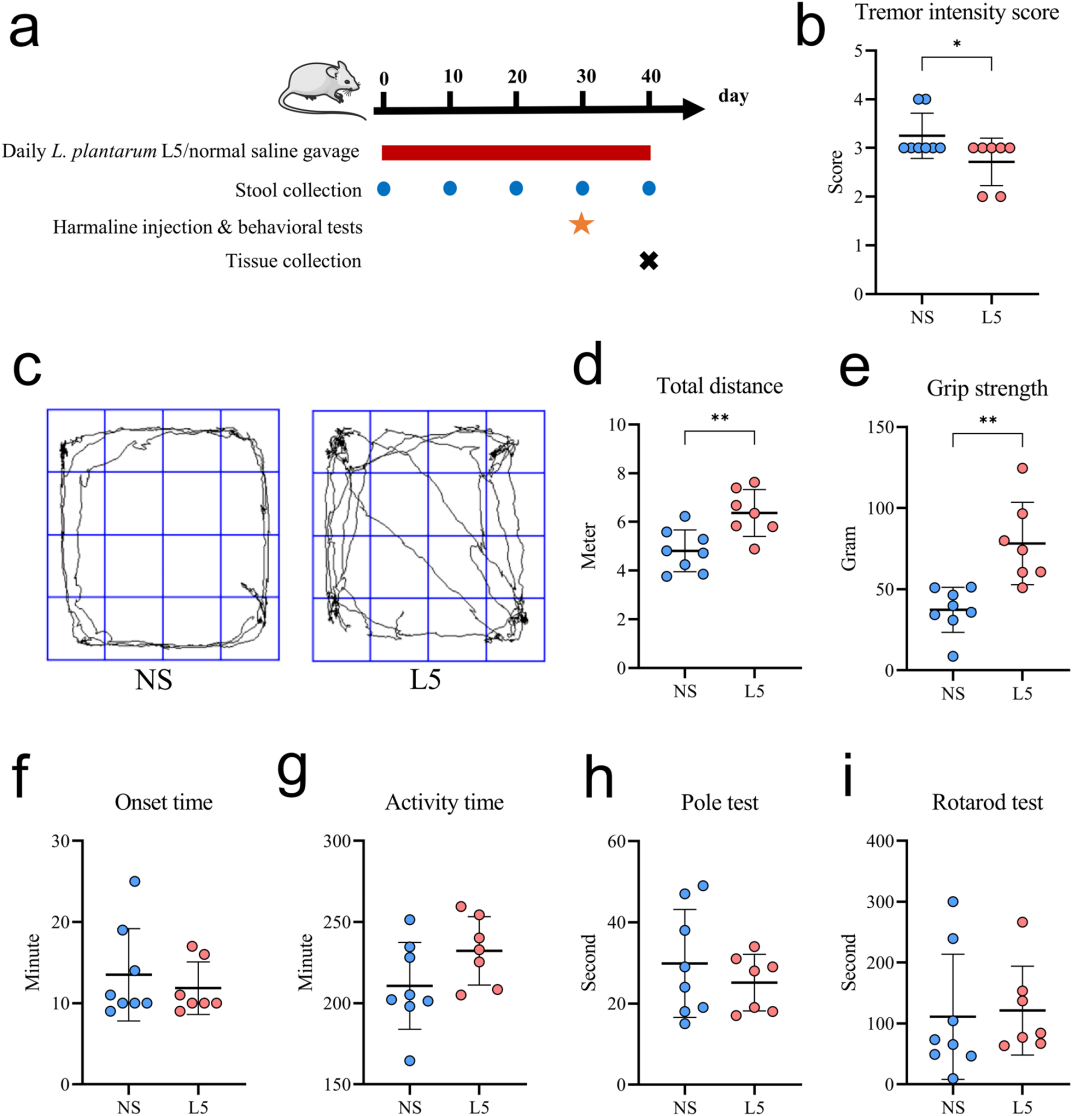
High-GABA-producing *L. plantarum* strain L5 ameliorated the harmaline-induced ET symptoms in mice. **a** Study design involving mice treated with L5 or normal saline daily for four weeks, followed by harmaline treatment to induce ET. **b** Quantitative analysis of the tremor intensity score. **c** Representative activity trajectories. **d** Total distance covered in 5 min. **e** Grip strength. **f** Onset time. **g** Active duration in 5 min. **h**, **i** Quantitative analysis of pole test (**h**) and rotarod test data (**i**). L5, *L. plantarum* L5; NS, normal saline. Data are presented as the mean ± standard deviation. ^*^P < 0.05; ^**^P < 0.01

Mice were administered with L5 or normal saline daily for four weeks; harmaline was used to induce ET, and behavioral tests were performed (Fig. 3a). Mice treated with L5 demonstrated a milder tremor intensity, longer total distance traveled, and a firmer grip strength than those treated with normal saline (Additional files 2 and 3: Videos and Fig. 3b–e). However, the results of activity time, pole test, and rotarod test showed no significant differences between the two groups (Fig. 3f–i). These results suggested that supplementation with high-GABA-producing *L. plantarum* L5 alleviated the harmaline-induced tremor symptoms in mice. In contrast, administering heat-killed L5 to mice yielded no discernible therapeutic effects on ET (Additional file 1: Fig. S3).

### *L. plantarum* L5 attenuated the CNS inflammation in ET mice

**Fig. 4:**
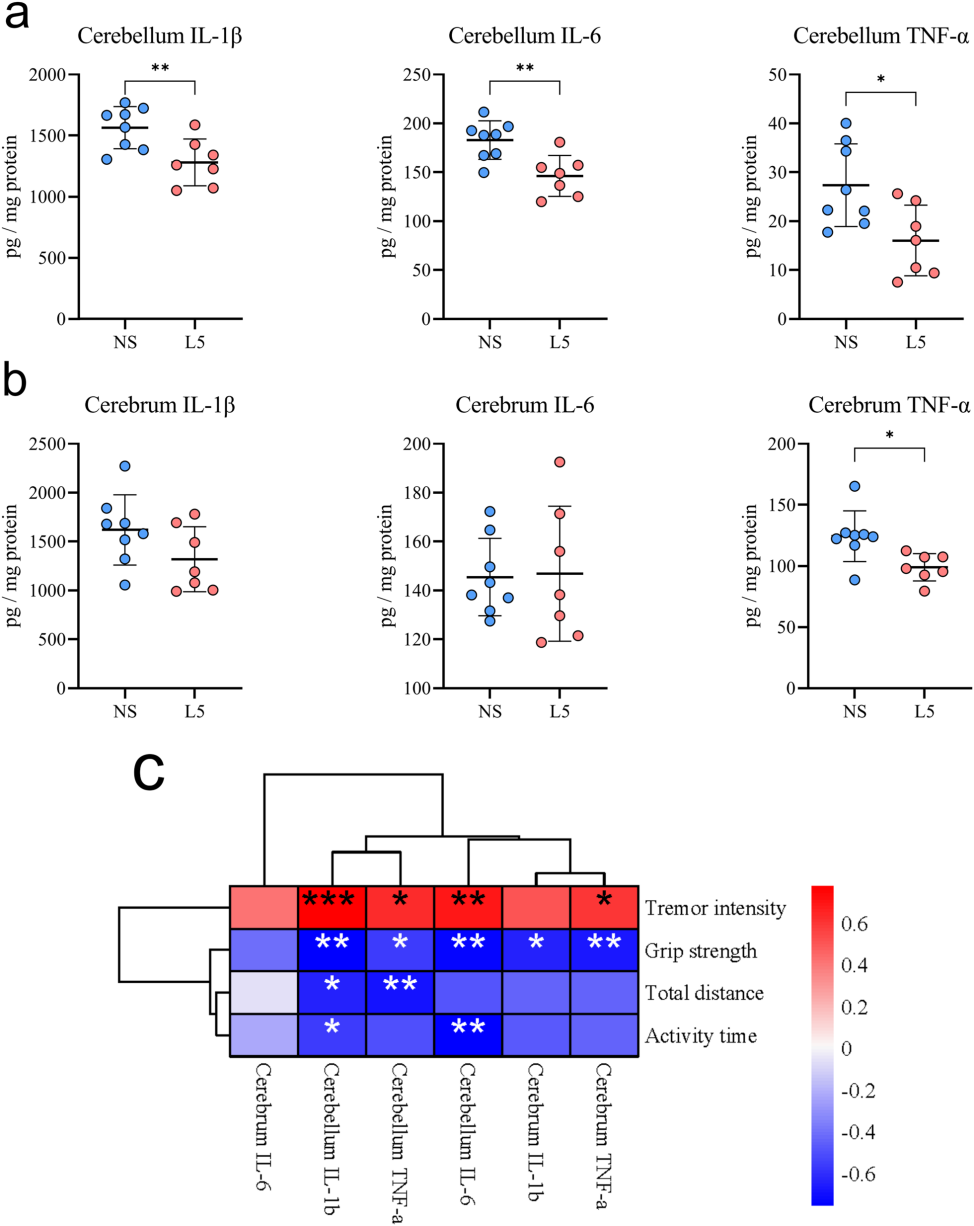
*L. plantarum* L5 attenuated the central nervous system inflammation in ET mice. **a, b** Concentrations of IL-1β, IL-6, and TNF-α in the cerebellum (**a**) and cerebrum (**b**) of mice with harmaline-induced ET treated with L5 or normal saline. **c** Heatmap showing correlations between the levels of inflammatory cytokines in the central nervous system with severity parameters of ET (tremor intensity, grip strength, total distance moved, and active duration). Data are presented as mean ± standard deviation. ^*^P < 0.05; ^**^P < 0.01; ^***^P < 0.001

Recent studies have suggested a link between ET and inflammation, especially inflammation in the CNS [28, 29]. Hence, the levels of pro-inflammatory cytokines in the cerebellum and cerebrum were assessed to determine the effects of L5 on CNS inflammation in ET mice. IL-1β, IL-6, and TNF-α in the cerebellum (Fig. 4a) and TNF-α in the cerebrum (Fig. 4b) were decreased significantly in the ET mice treated with L5 compared with those treated with normal saline. Moreover, these levels were correlated markedly with ET severity parameters, including tremor intensity, grip strength, total distance covered, and activity time (Fig. 4c). These findings suggested that L5 might ameliorate the tremors by attenuating CNS inflammation in the ET mice.

### *L. plantarum* L5 shaped the gut microbial composition and improved the microbial GABA-producing capacity in the small intestinal mucosa

**Fig. 5:**
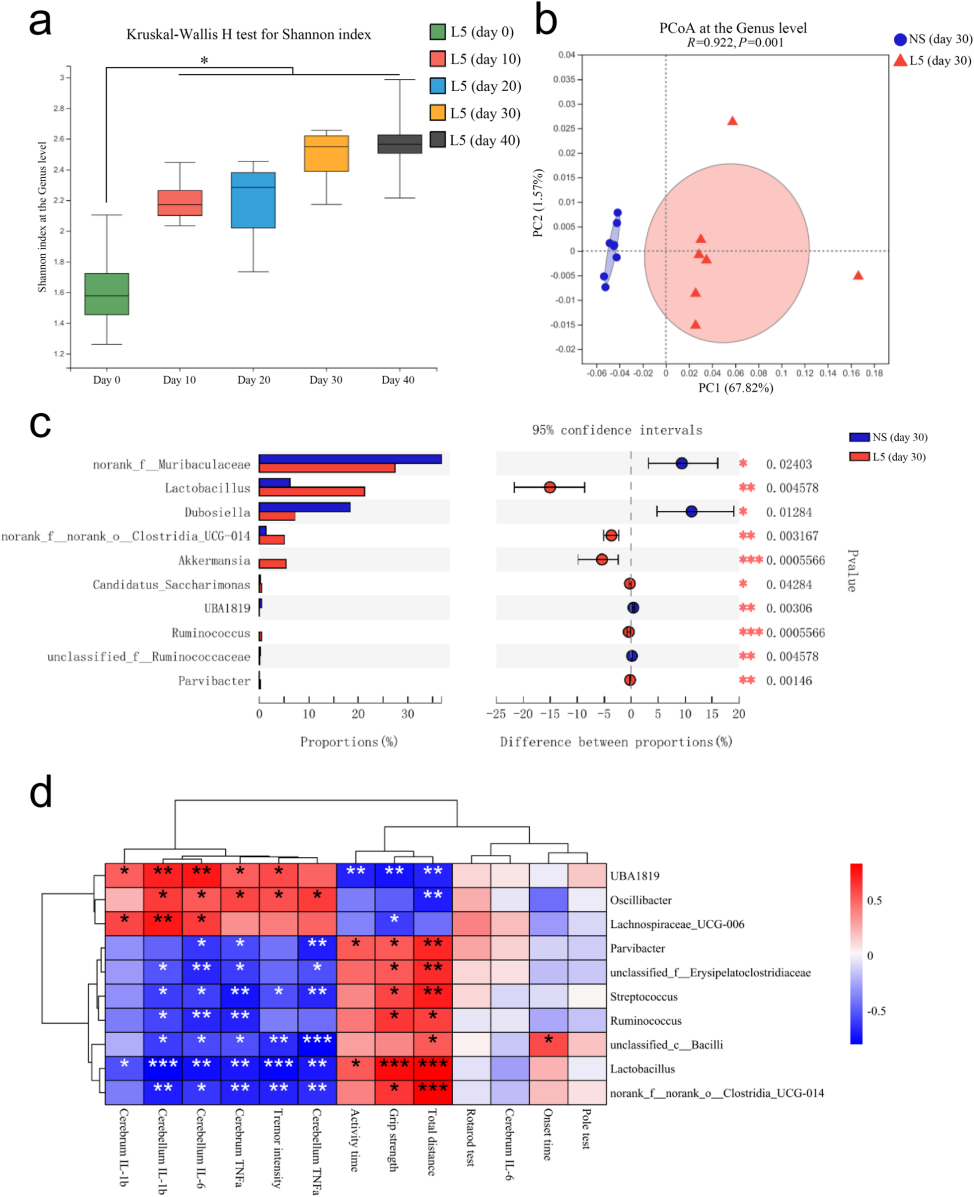
*L. plantarum* L5 shaped the gut microbial composition in ET mice. **a** Shannon’s diversity index at the generic level. **b** Principal coordinate analysis (PCoA) of microbiota composition at the generic level. **c** Bar plot of relative abundances of the top ten differential genera between ET mice treated with L5 or normal saline and the results of the Wilcoxon rank-sum test. **d** Heatmap of the correlations between the relative abundances of gut microbes at the generic level and ET severity parameters. L5, *L. plantarum* L5; NS, normal saline. Box plots indicate the median and interquartile range. ^*^P < 0.05; ^**^P < 0.01; ^***^P < 0.001

Microbes from fecal samples and the intestinal mucosa were analyzed using 16S rRNA gene sequencing to investigate the effects of L5 on the composition and function of gut microbiota in mice. The Shannon index indicated that the bacterial diversity gradually increased in mice during L5 administration (Fig. 5a). In addition, PCoA of the β-diversity demonstrated a noticeable difference in the composition of the gut microbiota between mice administered with L5 and those with normal saline (Fig. 5b). Moreover, the relative abundances of the genera *Lactobacillus* and *Akkermansia* (considered a therapeutic target in various neurological disorders [30]) were markedly enhanced in mice treated with L5 (Fig. 5c). Furthermore, the abundance of *Lactobacillus* in the fecal samples was negatively correlated with the severity of ET and the levels of pro-inflammatory cytokines in the CNS of ET mice (Fig. 5d).

**Fig. 6:**
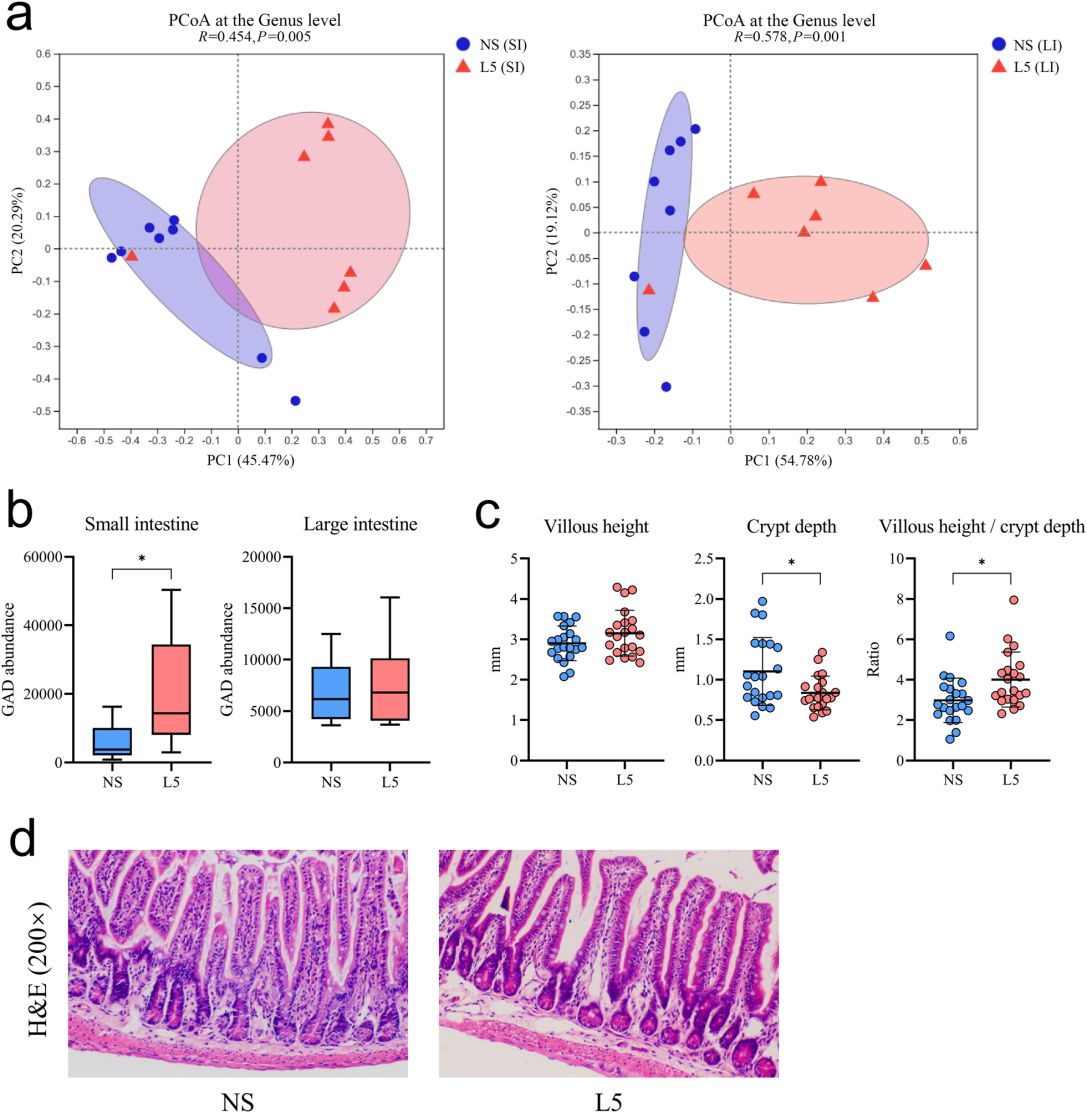
*L. plantarum* L5 increased the GABA-producing capacity of the small intestinal mucosa-resident microbes. **a** Principal coordinate analysis (PCoA) of the composition of small and large intestinal mucosa-associated microbiota at the generic level. **b** Glutamate decarboxylase levels in the small and large intestinal mucosa-associated microbiota, as predicted by PICRUSt2 analysis. **c** Villous height, crypt depth, and villous height-to-crypt depth ratio of the small intestine. **d** Representative hematoxylin and eosin staining of the small intestine. GAD, glutamate decarboxylase; L5, *L. plantarum* L5; LI, large intestine; NS, normal saline; SI, small intestine. Box plots indicating the median and interquartile range. Dot plots represent the mean ± standard deviation. ^*^P < 0.05

Recent studies have reported marked differences between the mucosa-associated microbiota and fecal microbiota [31, 32]. In addition, the mucosa-associated microbiota plays a more critical role in microbe–host interactions [33]. Therefore, the mucosa-associated microbiota from the small intestine and colon were also sequenced and compared between the mice administered with L5 and those with normal saline. PCoA plots revealed distinctly different small intestinal and colonic microbial compositions between the two groups of mice (Fig. 6a). Moreover, functional analysis using PICRUSt2 showed that the mice administered with L5 exhibited a higher *GAD* expression in the small intestinal mucosa-associated microbiota but not in the colonic mucosa-associated microbiota (Fig. 6b). L5 also reduced the crypt depth and increased the villous height-to-crypt ratio in the small intestine, improving intestinal development and digestive ability (Fig. 6c, d). These findings indicated that L5 could reshape the gut microbial composition, enhance the GABA-producing capacity of the mucosa-associated microbiota, and modulate the structure of the small intestinal barrier.

### *L. plantarum* L5 significantly increased GABA levels in the small intestine and cerebellum of ET mice

**Fig. 7:**
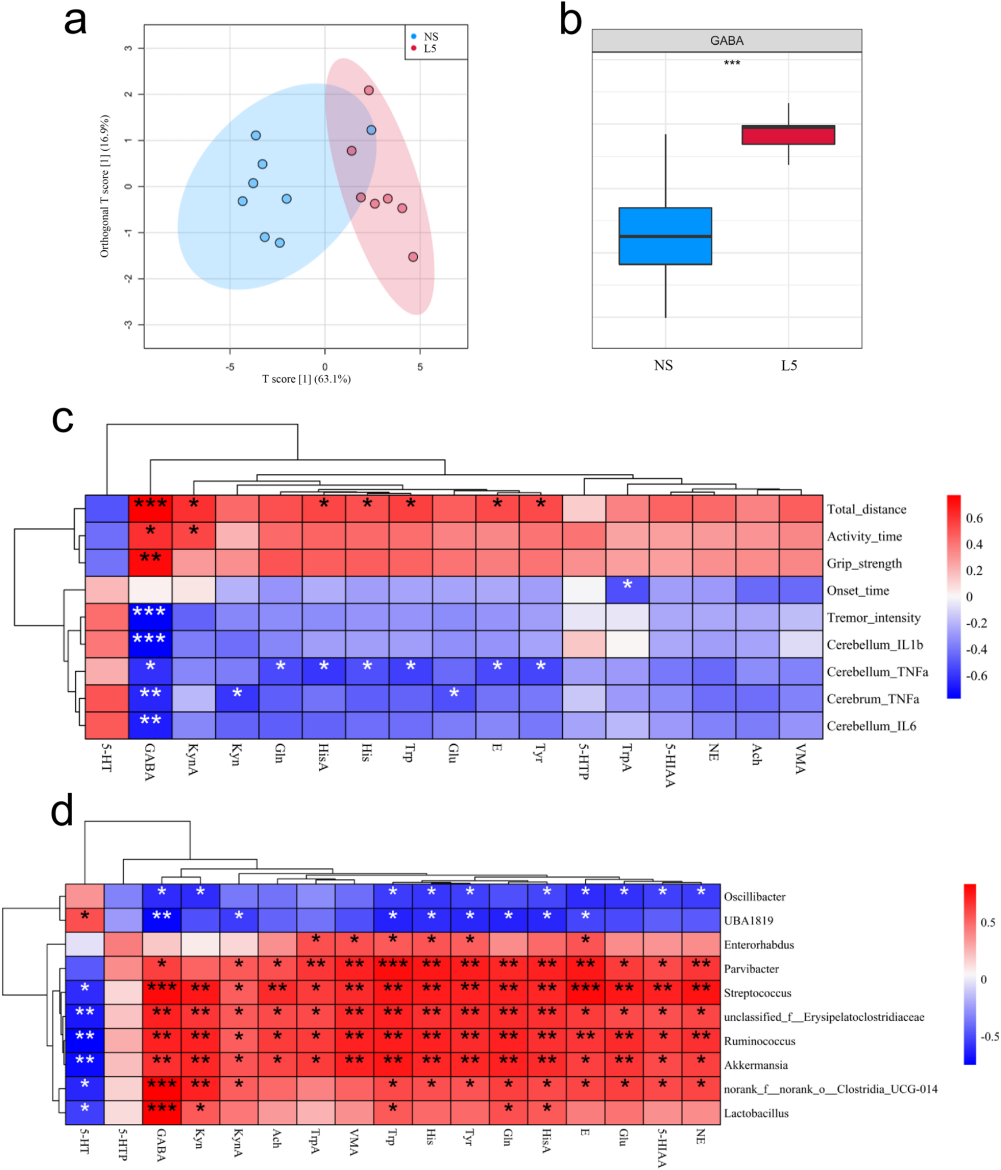
*L. plantarum* L5 enhanced the GABA levels in the small intestines of ET mice. **a** Orthogonal partial least-squares discriminant analysis (OPLS-DA) score plot of the neurotransmitter levels in the small intestine. **b** Relative levels of GABA in the small intestine. **c** Heatmap showing the correlations between the neurotransmitter levels in the small intestine and the parameters indicating ET severity. **d** Heatmap showing the correlations between the neurotransmitter levels in the small intestine and the generic-level abundances of gut microbes. 5-HIAA, 5-hydroxyindole-3-acetic acid; 5-HT, serotonin hydrochloride; 5-HTP, 5-hydroxytryptophan; Ach, acetylcholine chloride; E, adrenaline hydrochloride; GABA, γ-aminobutyric acid; Gln, L-glutamine; Glu, L-glutamic acid; His, L-histidine; HisA, histamine; Kyn, DL-kynurenine; KynA, kynurenic acid; L5, *L. plantarum* L5; NE, noradrenaline hydrochloride; NS, normal saline; Trp, L-tryptophan; TrpA, tryptamine; Tyr, L-tyrosine; TyrA, tyramine; VMA, vanillymandelic acid. The box plots indicate the median and interquartile range. ^*^P < 0.05; ^**^P < 0.01; ^***^P < 0.001

**Fig. 8:**
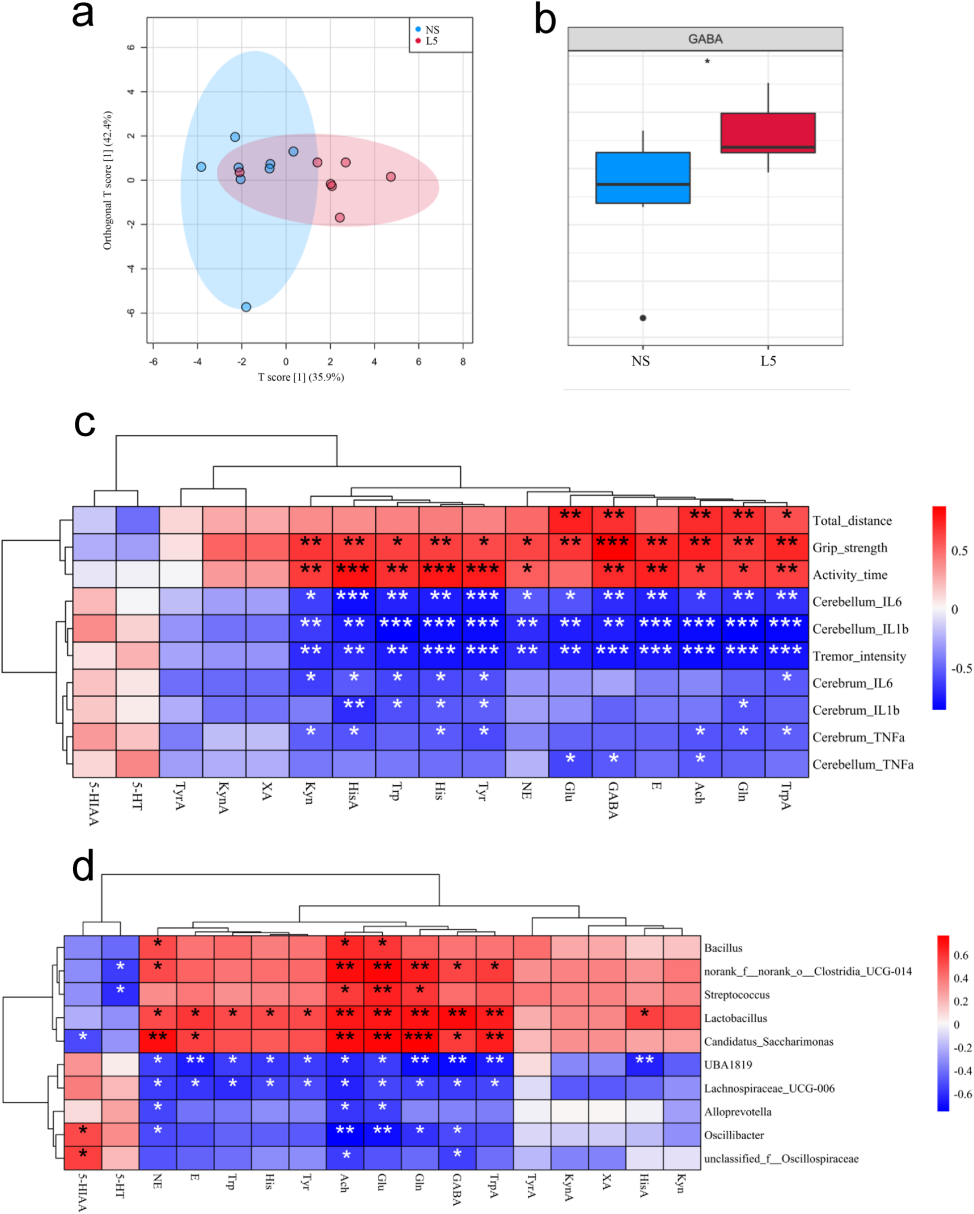
*L. plantarum* L5 elevated the level of GABA in the cerebellum of ET mice. **a** Orthogonal partial least-squares discriminant analysis (OPLS-DA) score plot of cerebellar neurotransmitter levels. **b** The relative level of cerebellar GABA. **c** Heatmap showing the correlations between the cerebellar neurotransmitter levels and the parameters indicating ET severity. **d** Heatmap indicating the correlations between the cerebellar neurotransmitter levels and generic-level abundances of gut microbes. 5-HIAA, 5-hydroxyindole-3-acetic acid; 5-HT, serotonin hydrochloride; Ach, acetylcholine chloride; E, adrenaline hydrochloride; GABA, γ-aminobutyric acid; Gln, L-glutamine; Glu, L-glutamic acid; His, L-histidine; HisA, histamine; Kyn, DL-kynurenine; KynA, kynurenic acid; L5, *L. plantarum* L5; NE, noradrenaline hydrochloride; NS, normal saline; Trp, L-tryptophan; TrpA, tryptamine; Tyr, L-tyrosine; TyrA, tyramine; XA, xanthurenic acid. The box plots indicate the median and interquartile range. ^*^P < 0.05; ^**^P < 0.01; ^***^P < 0.001

To determine the effects of L5 on the levels of neurotransmitters (including GABA) in ET mice, a targeted metabolomic analysis was performed to assess their levels in the small intestine, plasma, and cerebellum of ET mice administered with L5 or normal saline. The OPLS-DA score plots indicated global differences in the levels of neurotransmitters in the small intestine, plasma, and cerebellum between the two groups of mice (Fig. 7a and 8a; Additional file 1: Fig. S4a). Additionally, compared with mice receiving normal saline, the mice treated with L5 demonstrated a remarkable elevation in the GABA levels in the small intestine and cerebellum but not in the plasma (Fig. 7b, 8b; Additional file 1: Fig. S4b and S5a, b). Furthermore, correlation analyses revealed that the concentrations of GABA in the small intestine and cerebellum were negatively correlated with ET severity and positively correlated with the relative abundance of *Lactobacillus* (Fig. 7c,d; Fig. 8c,d; and Additional file 1: Fig. S4c, d). These results suggested that L5 alleviated the tremors in ET mice by increasing the levels of GABA in the small intestine and the cerebellum.

## Discussion

**Fig. 9:**
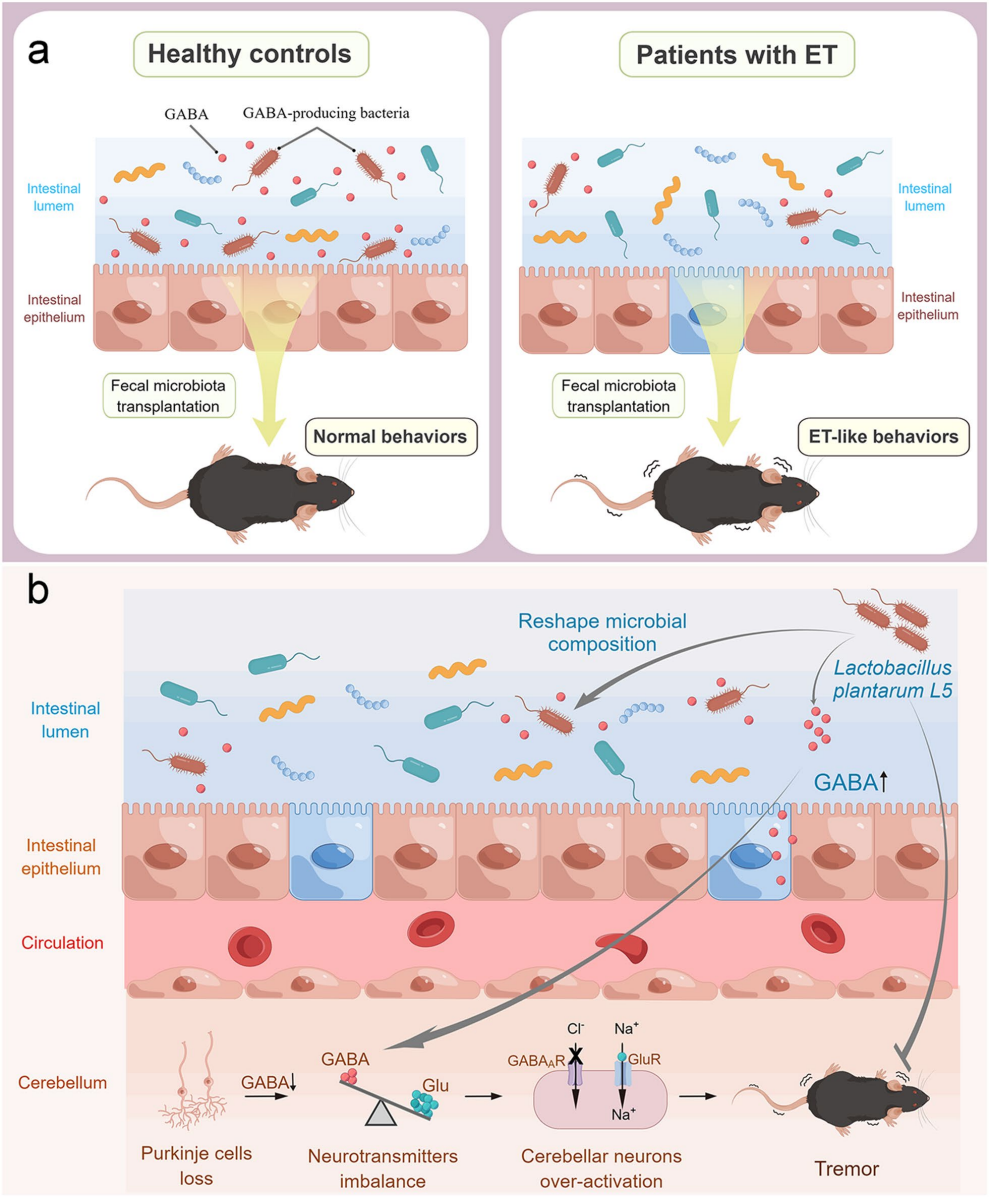
The gut microbiota in patients with ET has a lower GABA-producing capacity and is able to induce ET-like behavioral abnormalities in mice (**a**). Administration of *Lactobacillus plantarum* L5 alleviates ET by reshaping the gut microbial composition, increasing the GABA-producing capacity of the mucosa-associated microbiota, and elevating the concentration of GABA in the cerebellum (**b**)

In this study, we found that the gut microbiota of patients with ET had a lower GABA-producing capacity than that in the healthy controls, which induced prolonged tremors and impaired mobility in mice. Additionally, targeting the gut microbiota by administering the high-GABA-producing *L. plantarum* strain L5 significantly ameliorated the tremors in ET mice by reshaping the gut microbial composition, enhancing the GABA-producing capacity of the mucosa-associated microbiota, and elevating the GABA concentrations in the cerebellum (Fig. 9). To our knowledge, this is the first study to highlight that GABA-producing gut microbes play a crucial role in the pathogenesis of ET and, therefore, may have a therapeutic role.

The GABA hypothesis, which postulates noninhibition of deep cerebellar neurons due to reduced GABA generation, has mainly been used to explain the pathophysiological basis of ET [6]. First, degeneration of the GABAergic neurons in the cerebellum and a reduction in the concentrations of GABA in the cerebrospinal fluid were observed in patients with ET [7, 34]. Second, global knockout of the GABA_A_ receptor alpha-1 subunit was sufficient to produce ET-like postural and kinetic tremors in mice [35]. Third, several drugs that enhance GABA activity, such as gabapentin and topiramate, reduced tremors in patients with ET [36]. However, the reasons underlying the reduction of GABA in patients with ET remain unclear. Recent research has revealed that gut microbes communicate with the CNS by producing specific neurotransmitters, indicating that they are an essential source of GABA [23]. In this study, we observed a lower concentration of fecal GABA, decreased *GAD* expression in the fecal microbiota, and a positive correlation between the levels of GABA and *GAD* expression in patients with ET. These findings indicate that the lower GABA-producing capacity of the gut microbiota might lead to a decrease in the level of GABA and subsequently contribute to the development of ET.

An association between the gut microbiota and neurological diseases, such as Alzheimer’s and Parkinson’s diseases, has been widely reported [37]. Furthermore, a recent study analyzed the gut microbiota of 54 patients with ET and 54 normal controls using 16S rRNA gene sequencing and reported a lower species richness and altered composition of the gut microbiota in patients with ET [11]. Moreover, microbiome transplantation from patients with neurological disorders such as multiple sclerosis, autism, or stroke to SPF or germ-free mice accelerated and even triggered these diseases in them [37]. Similarly, in this study, transplantation of gut microbiota obtained from patients with ET led to prolonged tremors and impaired mobility in murine models of ET, indicating that the gut microbiota is involved in the initiation and progression of ET. Thus, gut microbiota holds excellent promise as a therapeutic target for ET.

Recent research highlighting the potential of microbe-based interventions for regulating gut dysbiosis-driven neurological disorders indicated that fecal microbiota transplantation and probiotics are promising therapeutic approaches [37]. A previous study by our research group revealed that regulating the gut microbiota via transplantation of fecal microbiota alleviated tremors in a patient with ET [12]. However, the clinical applicability of this procedure is limited as the composition of gut microbiota varies across healthy donors, and the incidence of adverse effects, such as death, infection, and fever, remains high [38]. Another microbe-based intervention—the administration of probiotics—has improved several neurological disorders by restoring the gut microbiota balance [37]. In this study, supplementation with L5 significantly elevated the levels of GABA in the small intestines and cerebellum, reduced the tremor severity, increased the movement capability, and enhanced the grasping ability of ET mice. Hence, L5 is a promising candidate for treating ET.

Despite the potential for gut microbial-driven neurotransmitters to reach the CNS through circulation or the vagus nerve [39], only small amounts of GABA in the blood can cross the blood–brain barrier [25]. Furthermore, GABA receptors are located throughout the enteric nervous system [40]. In our study, the supplementation of L5 did not lead to any conspicuous increase in the plasma concentrations of GABA in mice. Hence, it was hypothesized that the GABA synthesized by the intestinal L5 was conveyed to the brain via the “enteric nervous system–vagus nerve–brain” axis and subsequently relieved the tremor.

An accumulating body of evidence indicates that gut dysbiosis, characterized by a decreased abundance of probiotics and an overgrowth of pathobionts, can contribute to a leaky gut, which further results in neuroinflammation by elevating the levels of the metabolites produced by bacteria and the inflammatory cytokines in the CNS [39]. In this study, the administration of L5 significantly elevated the relative abundance of several probiotics (e.g., *Lactobacillus* and *Akkermansia*), improved the small intestinal barrier, and attenuated neuroinflammation in ET mice. These findings indicate the mechanisms of action of L5 in the treatment of ET, in addition to increasing GABA levels in the cerebellum.

This study has several limitations. First, although the patients with ET and healthy controls were strictly matched for age and sex when analyzing GABA concentrations in the feces and functions of the gut microbiota, the sample sizes were relatively small. Second, the patients with ET exhibited decreased intestinal GABA levels, while mice supplemented with L5 showed a notable increase in cerebellar GABA levels, suggesting a potential link between intestinal and cerebellar GABA. However, the concentrations of cerebellar GABA in patients with ET and the relationship between fecal and cerebellar GABA were not assessed. Third, a previous post-mortem study did not reveal any significant difference in the level of GABA receptors in the cerebellum of patients with ET [41], while another study demonstrated a decrease of GABA receptors [10]. Given the potential involvement of defective GABA receptors in the pathogenesis of ET, the effect of high-GABA-producing probiotics in improving ET in this type of patients remains to be confirmed by further clinical studies.

## Conclusions

The gut microbiota of patients with ET has a lower GABA-producing capacity and could induce ET-associated behavioral abnormalities in the murine ET model. Administration of *L. plantarum* L5 alleviates ET by reshaping the gut microbial composition, enhancing the GABA-producing capacity of the mucosa-associated microbiota, elevating the cerebellar GABA concentration, and decreasing inflammation in the CNS. Hence, L5 is a promising candidate for treating ET.

### Supplementary Information

**Additional file 1.** **Figure S1.** Administration of gut microbiota obtained from patients with ET led to a substantial alteration in the composition of gut microbiota and a notable decrease in the abundance of GABA-producing microbiota in mice. **Figure S2. **Oral administration of GABA fails to alleviate the severity or duration of tremors in murine ET. **Figure S3. **Administration of heat-killed *L. plantarum* L5 to mice did not yield any discernible therapeutic effects on ET. **Figure S4.** *L. plantarum *L5 altered the neurotransmitter levels in the plasma of ET mice. **Figure S5.** *L. plantarum* L5 altered the levels of neurotransmitters in the small intestines and cerebellum of ET mice. **Table S1.** Ion reactions for quantitative analysis of liquid chromatography-tandem mass spectrometry.** Table S2. **Demographic characteristics of patients with ET and healthy controls. **Table S3. **Clinical features of patients with ET. **Table S4. **The GABA-producing capacities of the 12 lactic acid bacteria strains containing *GAD*.

**Additional file 2.** Representative video of the behaviors of ET mice treated with normal saline.

**Additional file 3.** Representative video of the behaviors of ET mice treated with *L. plantarum* L5.

## Data Availability

The datasets generated and analysed in the current study are available in the NCBI repository with BioProject ID PRJNA904241 (https://dataview.ncbi.nlm.nih.gov/object/PRJNA904241) and PRJNA904560 (https://dataview.ncbi.nlm.nih.gov/object/PRJNA904560), in the article, and in its supplementary materials.

## Abbreviations

ASV: Amplicon sequence variant
CNS: Central nervous system
ELISA: Enzyme-linked immunosorbent assay
ET: Essential tremor
GABA: γ-Aminobutyric acid
IL: Interleukin
LAB: Lactic acid bacteria
MRS: Man-Rogosa-Sharpe
OPLS-DA: Orthogonal partial least-squares discriminant analysis
PCoA: Principal coordinate analysis
SPF: Specific-pathogen-free
TNF: Tumor necrosis factor
VIP: Variable importance in projection
