# Supplementary material for: Supplementation with high-GABA-producing Lactobacillus plantarum L5 ameliorates essential tremor triggered by decreased gut bacteria-derived GABA
Source: Transl Neurodegener. 2023 Dec 13;12:58. doi: 10.1186/s40035-023-00391-9 (PMC10717605; doi:10.1186/s40035-023-00391-9)
Supplement: Supplementary file 1 — Additional file 1. Figure S1. Administration of gut microbiota obtained from patients with ET led to a substantial alteration in the composition of gut microbiota and a notable decrease in the abundance of GABA-producing microbiota in mice. Figure S2. Oral administration of GABA fails to alleviate the severity or duration of tremors in murine ET. Figure S3. Administration of heat-killed L. plantarum L5 to mice did not yield any discernible therapeutic effects on ET. Figure S4. L. plantarum L5 altered the neurotransmitter levels in the plasma of ET mice. Figure S5. L. plantarum L5 altered the levels of neurotransmitters in the small intestines and cerebellum of ET mice. Table S1. Ion reactions for quantitative analysis of liquid chromatography-tandem mass spectrometry. Table S2. Demographic characteristics of patients with ET and healthy controls. Table S3. Clinical features of patients with ET. Table S4. The GABA-producing capacities of the 12 lactic acid bacteria strains containing GAD. [file 40035_2023_391_MOESM1_ESM.docx]

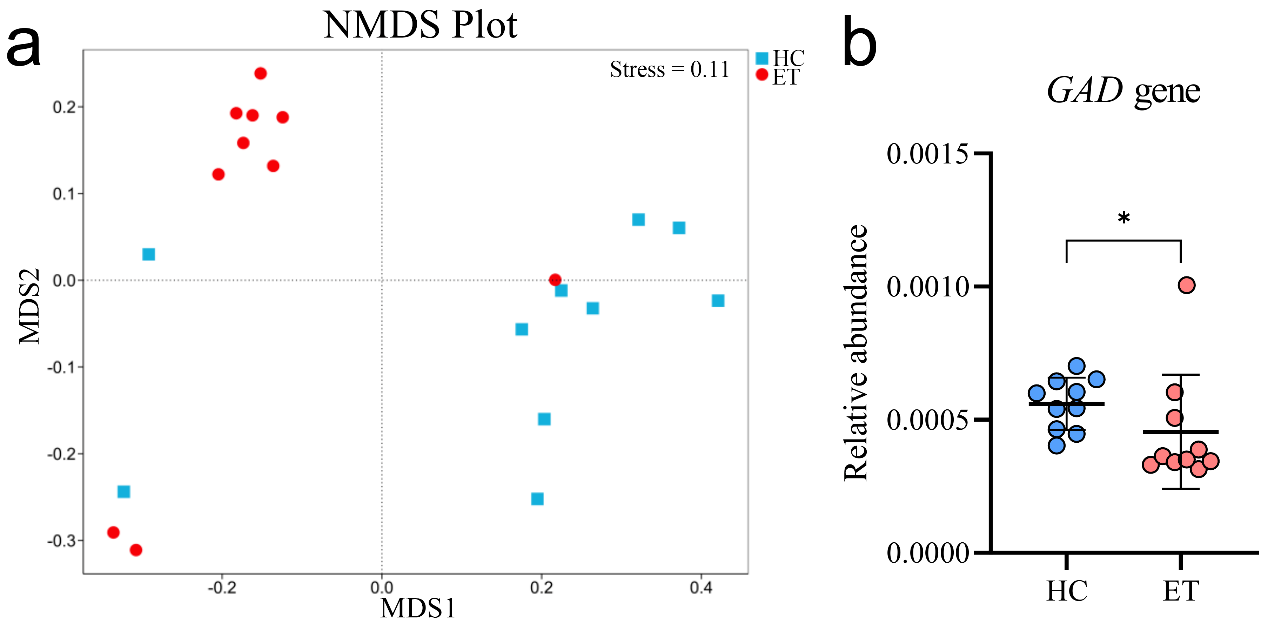


**Figure S1. Administration of gut microbiota obtained from patients with ET led to a substantial alteration in the composition of gut microbiota and a notable decrease in the abundance of GABA-producing microbiota in mice.** (a) Non-metric multidimensional scaling (NMDS) plots of the composition of the microbiota at the generic level. (b) Prediction of the relative abundance of microbiota containing *GAD*. Data are presented as the mean ± standard deviation. ^*^*P* < 0.05.


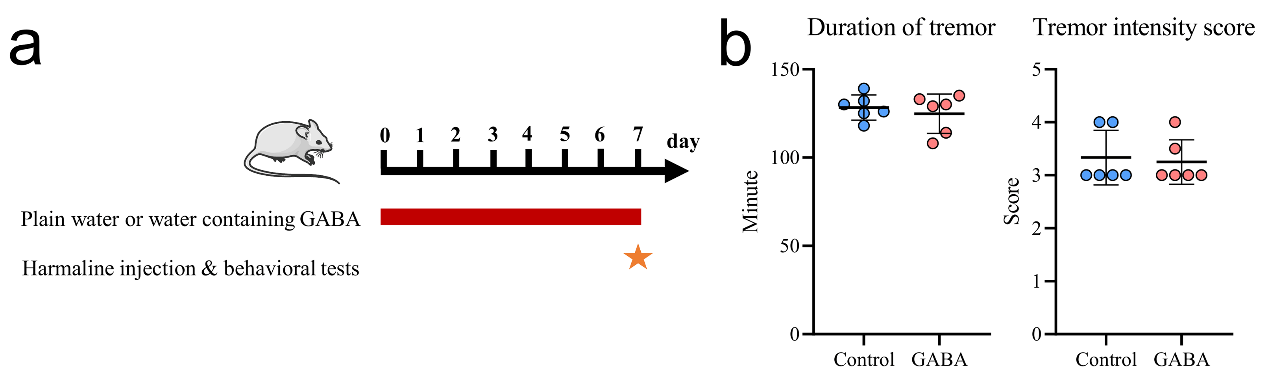


**Figure S2. Oral administration of GABA fails to alleviate the severity or duration of tremors in murine ET.** (a) The study design involved mice with either plain water (control) or water supplemented with 2 mg/mL GABA for seven days, followed by harmaline treatment to induce ET. (b) Quantitative analysis of the duration and severity of tremors. Data are presented as the mean ± standard deviation.


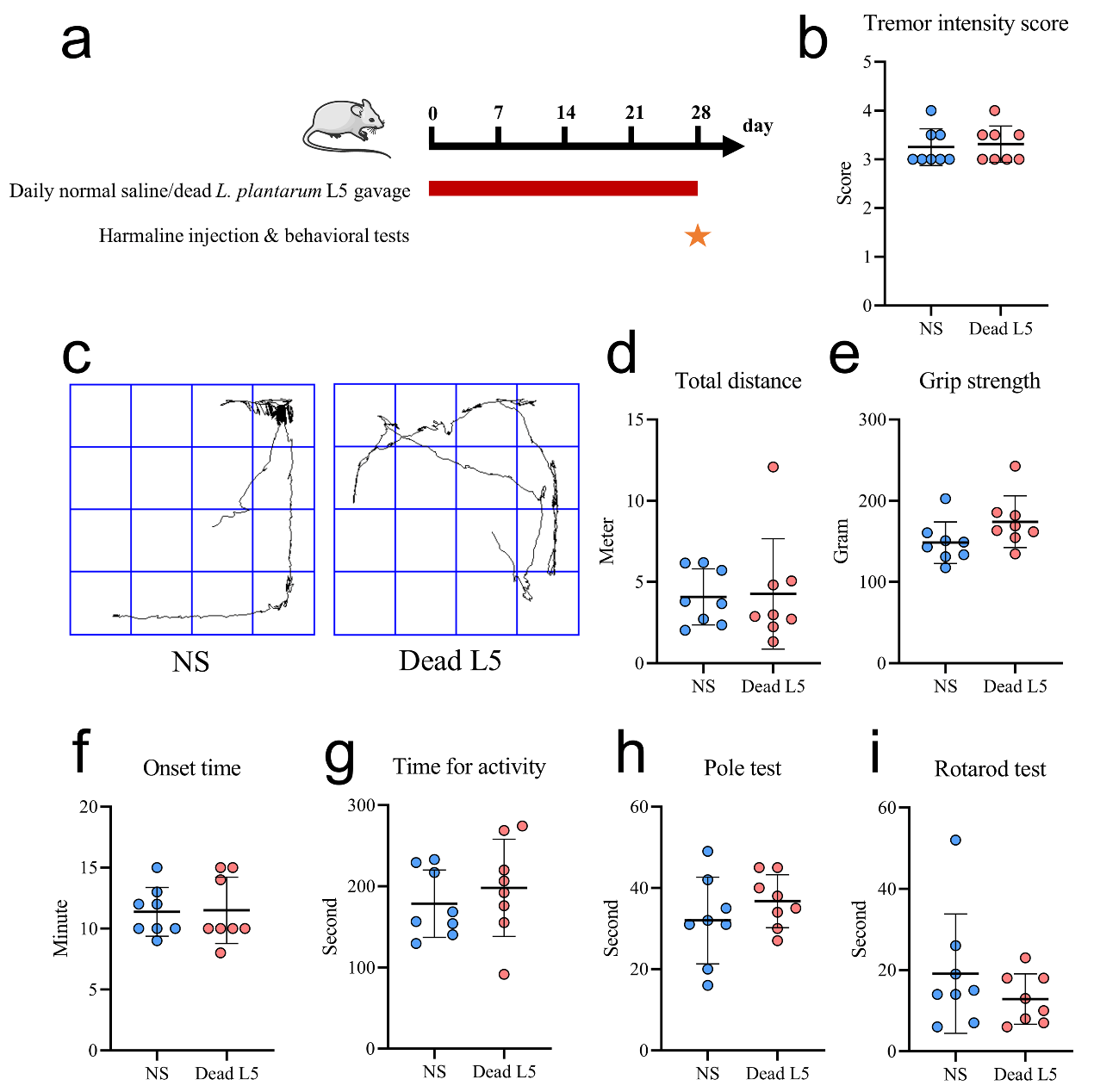


**Figure S3. Administration of heat-killed *L. plantarum* L5 to mice did not yield any discernible therapeutic effects on ET.** (a) Study design involving mice treated with normal saline or dead L5 daily for four weeks, followed by harmaline treatment to induce ET. (b) Quantitative analysis of the tremor intensity score. (c) Representative activity trajectories. (d) Total distance covered in 5 min. (e) Grip strength. (f) Onset time. (g) Active duration in 5 min. Quantitative analysis of the (h) pole test and (i) rotarod test data. L5, *L. plantarum* L5; NS, normal saline. Data are presented as the mean ± standard deviation.


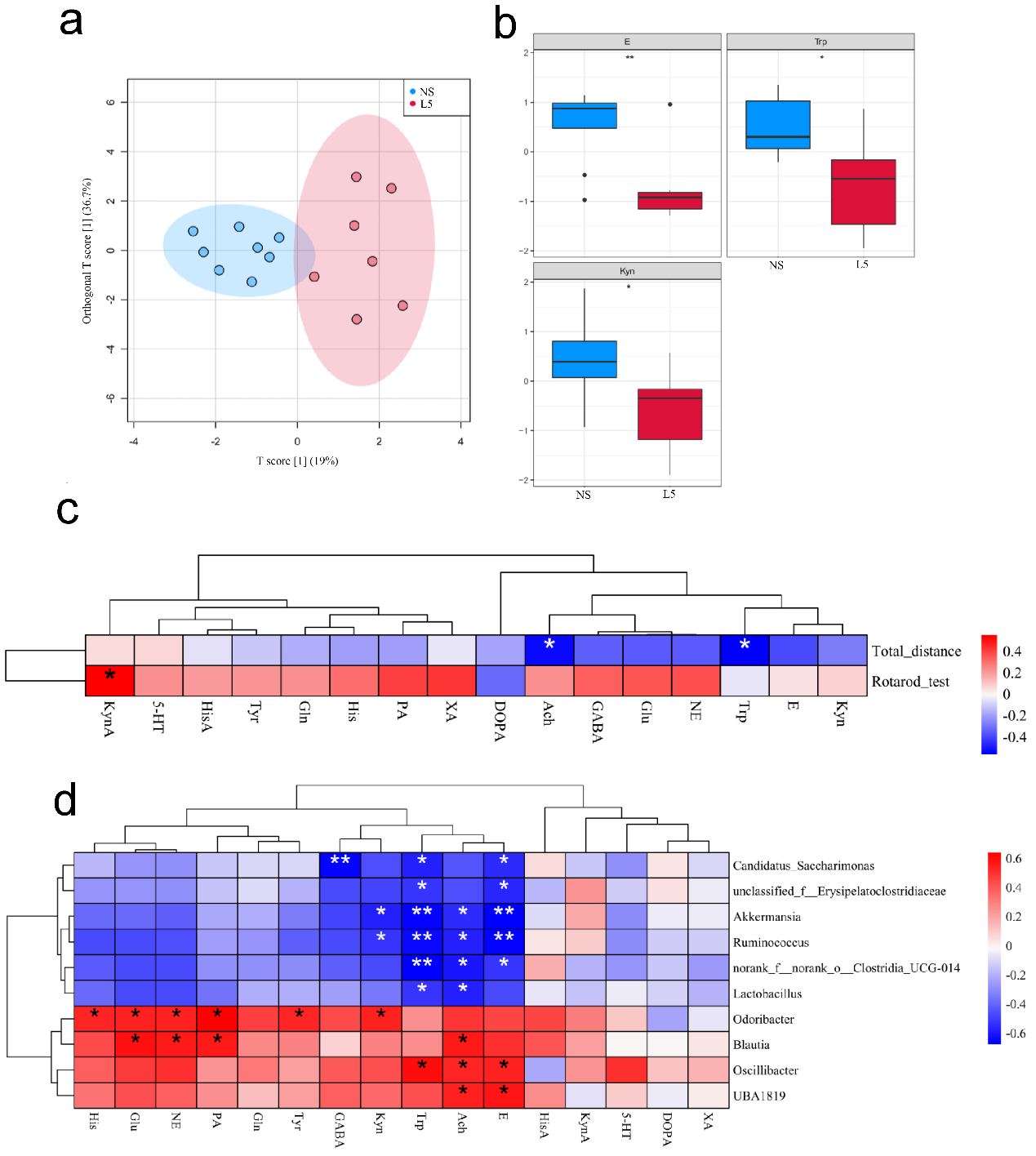


**Figure S4. *L. plantarum* L5 altered the neurotransmitter levels in the plasma of ET mice.** (a) Orthogonal partial least-squares discriminant analysis (OPLS-DA) score plot of plasma neurotransmitter levels. (b) Relative levels of differential neurotransmitters in the plasma of ET mice treated with L5 or normal saline. (c) Heatmap showing the correlations between the neurotransmitter levels in the plasma and ET severity parameters. (d) Heatmap indicating the correlations between the neurotransmitter levels in the plasma and generic-level abundances of gut microbes. 5-HT, serotonin hydrochloride; Ach, acetylcholine chloride; DOPA, levodopa; E, adrenaline hydrochloride; GABA, γ-aminobutyric acid; Gln, L-glutamine; Glu, L-glutamic acid; His, L-histidine; HisA, histamine; Kyn, DL-kynurenine; KynA, kynurenic acid; L5, *L. plantarum* L5; NE, noradrenaline hydrochloride; NS, normal saline; PA, picolinic acid; Trp, L-tryptophan; Tyr, L-tyrosine; XA, xanthurenic acid. Box plots indicate the median and interquartile range. ^*^*P* < 0.05; ^**^*P* < 0.01.


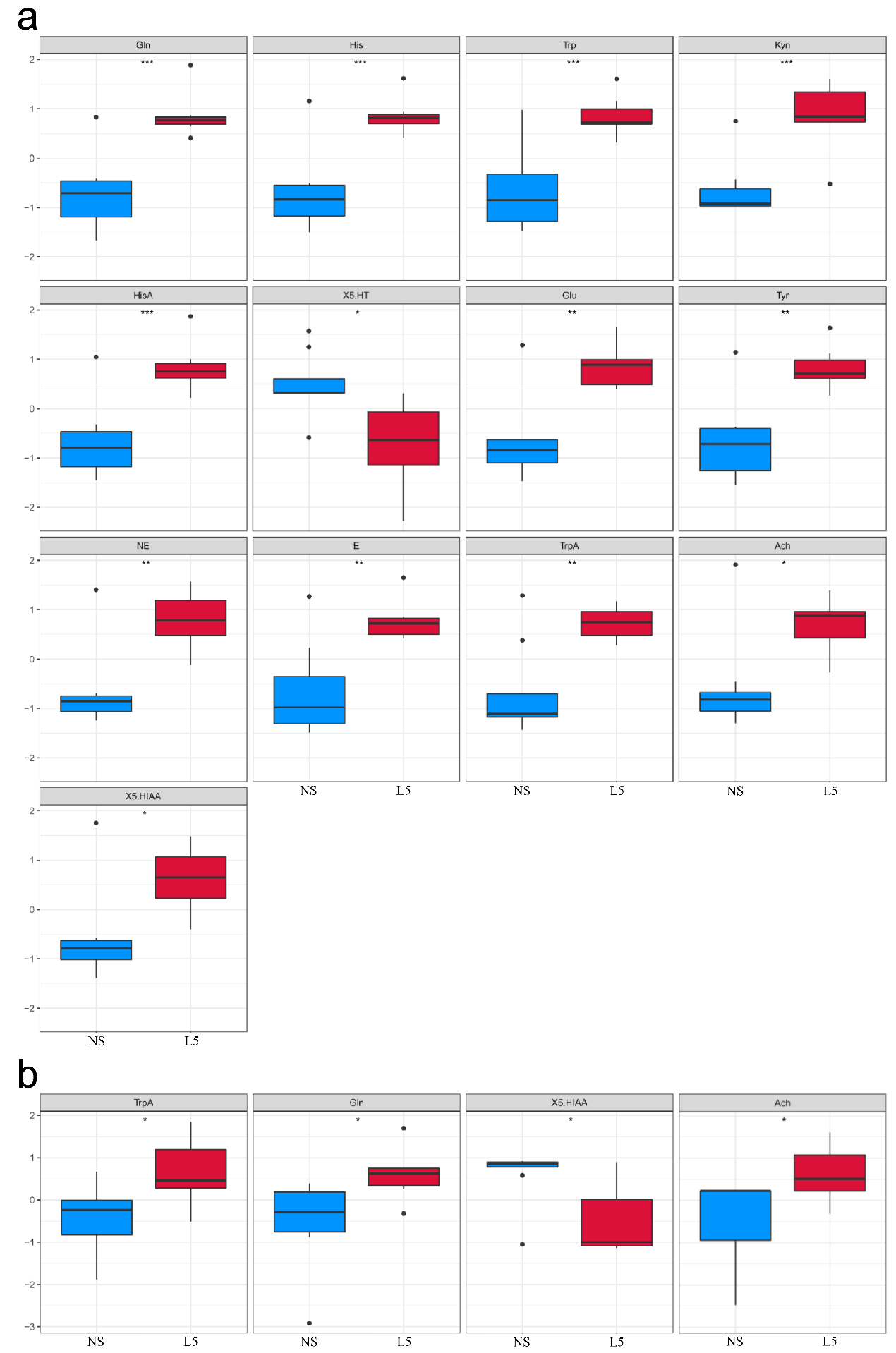


**Figure S5. *L. plantarum* L5 altered the levels of neurotransmitters in the small intestines and cerebellum of ET mice.** Relative levels of the different neurotransmitters (except GABA) in the (a) small intestine and (b) cerebellum of ET mice treated with L5 or normal saline. 5-HIAA, 5-hydroxyindole-3-acetic acid; 5-HT, serotonin hydrochloride; Ach, acetylcholine chloride; E, adrenaline hydrochloride; Gln, L-glutamine; Glu, L-glutamic acid; His, L-histidine; HisA, histamine; Kyn, DL-kynurenine; NE, noradrenaline hydrochloride; Trp, L-tryptophan; TrpA, tryptamine; Tyr, L-tyrosine. Box plots indicate the median and interquartile range. ^*^*P* < 0.05; ^**^*P* < 0.01; ^***^*P* < 0.001.

**Table S1. Ion reactions for quantitative analysis of liquid chromatography-tandem mass spectrometry.**

| Component Name | Abbreviation | Precursor (m/z) | Product (m/z) | Declustering potential (V) | Entrance potential (V) | Collision energy (eV) | Cell exit potential (V) |
| --- | --- | --- | --- | --- | --- | --- | --- |
| Histamine | HisA | 112.1 | 95 | 45 | 10 | 19 | 11 |
| Picolinic acid | PA | 124 | 78 | 28 | 10 | 24 | 8 |
| Tyramine | TyrA | 137.9 | 121 | 47 | 10 | 14 | 14 |
| Acetylcholine chloride | Ach | 146.1 | 87.1 | 50 | 10 | 19 | 10 |
| Hydroxytyramine hydrochloride | DA | 154.1 | 137.1 | 35 | 10 | 15 | 16 |
| Tryptamine | TrpA | 161.1 | 144 | 50 | 10 | 13 | 17 |
| Noradrenaline hydrochloride | NE | 170.2 | 152.1 | 30 | 10 | 11 | 8 |
| Serotonin hydrochloride | 5-HT | 177.1 | 160.1 | 45 | 10 | 15 | 15 |
| Adrenaline hydrochloride | E | 184.1 | 166.2 | 30 | 10 | 13 | 30 |
| Kynurenic acid | KynA | 190.1 | 144 | 60 | 10 | 25 | 17 |
| 5-Hydroxyindole-3-acetic acid | 5-HIAA | 191.9 | 146 | 40 | 10 | 14 | 18 |
| Levodopa | DOPA | 198.1 | 152 | 35 | 10 | 18 | 17 |
| Xanthurenic acid | XA | 206 | 178.1 | 70 | 10 | 22 | 10 |
| DL-Kynurenine | Kyn | 209 | 94 | 10 | 10 | 19 | 11 |
| Vanillymandelic acid | VMA | 221.1 | 203.1 | 60 | 10 | 11 | 13 |
| 5-Hydroxytryptophan | 5-HTP | 221.1 | 204 | 13 | 10 | 15 | 12 |
| Melatonine | MT | 233.2 | 174.1 | 50 | 10 | 19 | 20 |
| 4-Aminobutyric acid | GABA | 104.1 | 87.1 | 40 | 10 | 15 | 10 |
| L-Glutamine | Gln | 147.1 | 84.1 | 25 | 10 | 23 | 9 |
| L-Glutamic acid | Glu | 148.1 | 84.1 | 20 | 10 | 21 | 10 |
| L-Histidine | His | 156 | 110 | 50 | 10 | 19 | 12 |
| L-Tyrosine | Tyr | 182.1 | 165 | 40 | 10 | 13 | 20 |
| L-Tryptophan | Trp | 205.1 | 187.9 | 40 | 10 | 14 | 10 |
| Tryptophan-d3 | Trp-d3 | 208.2 | 146.9 | 40 | 10 | 24 | 18 |

**Table S2. Demographic characteristics of patients with ET and healthy controls.**

| Patients with ET  (*n* = 5) | Healthy controls  (*n* = 5) | Age (years) | Sex |
| --- | --- | --- | --- |
| Patient 1 | Healthy control 1 | 55 | Male |
| Patient 2 | Healthy control 2 | 57 | Female |
| Patient 3 | Healthy control 3 | 66 | Male |
| Patient 4 | Healthy control 4 | 31 | Male |
| Patient 5 | Healthy control 5 | 25 | Male |

ET, essential tremor.

**Table S3. Clinical features of patients with ET.**

| Patients with ET | Clinical features |
| --- | --- |
| Patient 1 | **Duration:** 6 years  **Tremor characteristics:** postural tremors of the upper limbs and head  **Other neurological signs:** none  **Treatment:** none |
| Patient 2 | **Duration:** > 20 years  **Tremor characteristics:** kinetic tremors of upper limbs  **Other neurological signs:** none  **Treatment:** topiramate and alprazolam |
| Patient 3 | **Duration:** > 10 years  **Tremor characteristics:** kinetic tremors of upper limbs  **Other neurological signs:** none  **Treatment:** oxiracetam |
| Patient 4 | **Duration:** 15 years  **Tremor characteristics:** kinetic tremors of upper limbs  **Other neurological signs:** none  **Treatment:** propranolol |
| Patient 5 | **Duration:** > 10 years  **Tremor characteristics:** kinetic tremors of upper limbs  **Other neurological signs:** none  **Treatment:** none |

**Table S4. The GABA-producing capacities of the 12 lactic acid bacteria strains containing GAD.**

| Strains | GABA concentration in the MRS broth culture (mg/L) |
| --- | --- |
| *Lactobacillus plantarum L5* | 262.3±5.4 |
| *Lactobacillus plantarum L30* | 260.1±4.7 |
| *Lactobacillus brevis SR52-2* | 245.3±4.2 |
| *Lactobacillus plantarum SM14-6* | 218.6±5.3 |
| *Lactobacillus brevis ST4-6* | 216.4±12.8 |
| *Lactobacillus plantarum SM26-3* | 182.6±4.7 |
| *Lactobacillus paracasei R64* | 178.3±3.5 |
| *Lactobacillus brevis ST14-6* | 163.5±17.8 |
| *Lactobacillus paracasei Q17* | 161.7±5.2 |
| *Lactobacillus paraplantarum SR57-4* | 158.5±3.2 |
| *Lactobacillus plantarum SJ28-3* | 114.3±4.5 |
| *Lactobacillus plantarum SJ28-6* | 106.7±5.1 |

GABA, γ-aminobutyric acid; GAD, glutamate decarboxylase; MRS, De Man-Rogosa-Sharpe.
